# Impact of Sarcopenia on Outcomes Following Endovascular Treatment of Patients with Pelvic Venous Insufficiency

**DOI:** 10.1007/s00270-025-04051-5

**Published:** 2025-05-23

**Authors:** Sinan Sozutok, Ferhat Can Piskin, Omer Kaya, Hasan Bilen Onan, Huseyin Tugsan Balli, Erol Huseyin Aksungur

**Affiliations:** Faculty of Medicine, Department of Radiology, Cukurova University, Adana, Turkey

**Keywords:** Pelvic venous insufficiency, Endovascular embolization, Sarcopenia

## Abstract

**Purpose:**

To investigate the influence of sarcopenia on the outcomes of endovascular treatment in patients with pelvic venous insufficiency (PVI).

**Material and Methods:**

A retrospective analysis was conducted on 62 female patients who underwent endovascular treatment for PVI between January 2012 and July 2020. The patients were evaluated using the Visual Analog Scale (VAS) for chronic pelvic pain, both before treatment and three months post-treatment. A successful treatment outcome was defined as a reduction in pelvic pain by 50% or more. Sarcopenia was diagnosed based on the skeletal muscle mass index (SMI) of the patients.

**Results:**

Sarcopenia was present in 20 (32.3%) patients. There was no significant difference in preprocedural VAS scores between sarcopenic and non-sarcopenic patients (36.2 ± 16.9 vs. 34.9 ± 16.1, *p* = 0.781). However, non-sarcopenic patients achieved a significantly greater reduction in VAS scores post-treatment (67.6 ± 22.4% vs. 53.1 ± 24.4%, *p* = 0.025). A moderate negative correlation was observed between sarcopenia and successful treatment outcomes (*r* = -0.365, *p* = 0.004).

**Conclusion:**

Sarcopenia negatively impacts the effectiveness of endovascular treatment in patients with PVI.

**Graphical Abstract:**

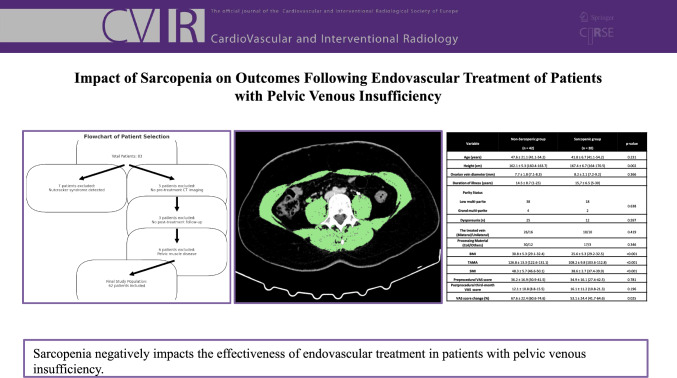

## Introduction

Chronic pelvic pain (CPP) is defined as cyclic or non-cyclic pain in women, typically involving the entire abdomen, particularly the pelvic region, and persisting for at least six months [1]. The incidence of CPP among women aged 18–50 years has been reported to range from 2.1 to 24% [1, 2]. CPP can result from various gynecological conditions, including endometriosis, uterine fibroids, adenomyosis, and inflammatory processes [1, 2].

Pelvic venous insufficiency (PVI) has been recognized as one of the leading causes of CPP. First described by Taylor in 1949, PVI is characterized by pelvic, vulvar, or perineal varicose veins, accompanied by symptoms such as pain, pelvic fullness, dyspareunia, and urgency [3–5]. In recent years, endovascular treatment has emerged as a crucial therapeutic option for PVI. However, treatment outcomes vary significantly among patients, and it remains unclear which patients are most likely to benefit from endovascular intervention [6, 7].

Sarcopenia, a clinical condition frequently observed in the elderly and individuals with chronic diseases, is characterized by the progressive loss of muscle mass and function [8]. This condition is commonly associated with poor dietary habits and a sedentary lifestyle [9]. Sarcopenia is more than a simple loss of muscle mass; it is a significant clinical concern that can lead to diminished cardiopulmonary performance and an increased risk of mortality due to associated metabolic disorders [10]. Studies have indicated that the prevalence of sarcopenia ranges from 5 to 13% in individuals under 60 years of age, with a marked increase to nearly 50% in those over 80 years [11–13]. The decline in muscle mass accelerates with advancing age, particularly after 50 years, and this trend is more pronounced in women than in men [14].

Sarcopenia is also associated with chronic pain and limited physical activity, which are not only potential contributors to the persistence of pelvic venous insufficiency symptoms but may also reduce the effectiveness of endovascular treatment in PVI patients [15]. Based on these associations, our hypothesis is that sarcopenia negatively impacts the outcomes of embolization therapy in PVI patients by impairing recovery and limiting the procedural benefits through decreased physical capacity and systemic effects.

Therefore, this study aims to investigate the influence of sarcopenia on the outcomes of endovascular treatment in patients with PVI.

## Materials and Methods

This study retrospectively analyzed data from 62 female patients who underwent endovascular ovarian vein embolization for pelvic venous insufficiency (PVI) between January 2012 and July 2020. The study protocol was approved by the Çukurova University Faculty of Medicine Clinical Research Ethics Committee (decision number 5/2021, 116th meeting).

The included patients presented with chronic pelvic pain (CPP) and pelvic varicose veins and were diagnosed with PVI based on evaluations conducted using intravenous (IV) contrast-enhanced computed tomography (CT). These patients subsequently underwent endovascular treatment in the interventional radiology unit of our hospital.

**Fig. 1:**
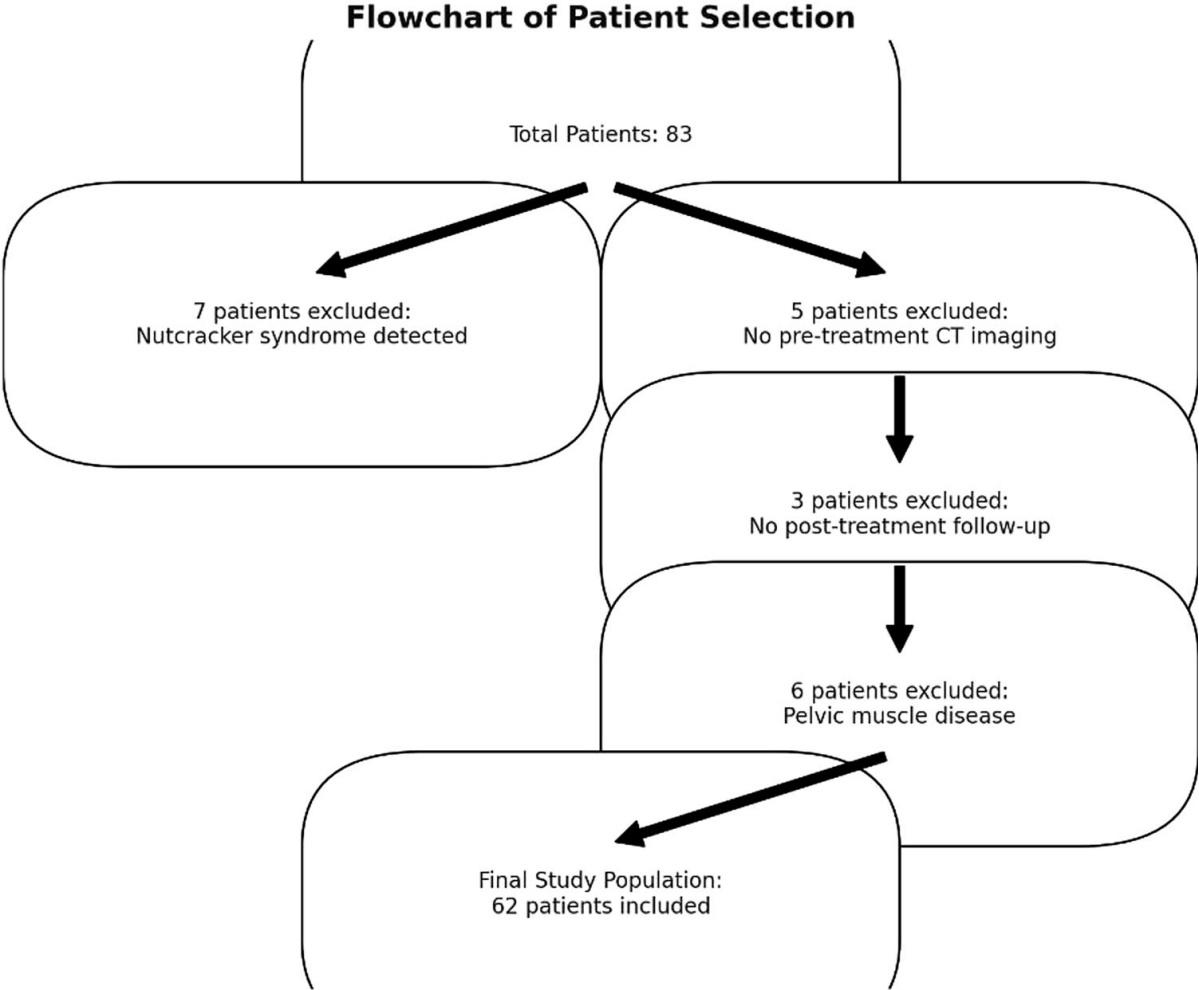
Flowchart depicting the study design and patient selection process

Exclusion criteria included secondary causes of PVI such as May-Thurner syndrome or Nutcracker syndrome, confirmed through clinical evaluation or imaging. Additionally, patients with other diseases causing muscle dysfunction, including pelvic myofascial syndrome, were excluded following assessments conducted in collaboration with physiatrists and vascular specialists. Patients who did not attend follow-up visits or had incomplete post-treatment data were also excluded, as well as those under the age of 18 (Fig. 1).

### Diagnosis of Pelvic Venous Congestion Syndrome

Initially, transabdominal pelvic venous Doppler ultrasonography (US) was performed on patients with CPP and pelvic varicose veins. Patients exhibiting pelvic venous reflux were further evaluated with IV contrast-enhanced CT, and those with an ovarian vein diameter of ≥ 6 mm were diagnosed with PVI. Patients with clinical findings consistent with PVI and pelvic varices but without ovarian vein enlargement detected on CT underwent venography to confirm the diagnosis. This step ensured accurate diagnosis in cases where non-invasive imaging findings were inconclusive.

The selected patient population was defined anatomically in accordance with the Society for Vascular Surgery and American Venous Forum classification system. This classification divides PVI into categories based on the affected venous territories, including the ovarian veins, internal iliac veins, and other pelvic venous tributaries [3].

### Measurement of Muscle Area

Muscle area measurements were conducted using OsiriX software (version 12.0, OsiriX Foundation, Geneva, Switzerland). All measurements were performed by a single experienced abdominal radiologist. The cross-sectional area was analyzed on a single CT slice passing through the upper endplate of the L3 vertebra. The total abdominal muscle area, including the psoas, erector spinae, quadratus lumborum, transversus abdominis, external and internal obliques, and rectus abdominis, was segmented using a threshold density range of − 29 to + 150 Hounsfield units. Faulty muscle tissue boundaries were manually corrected.

**Fig. 2:**
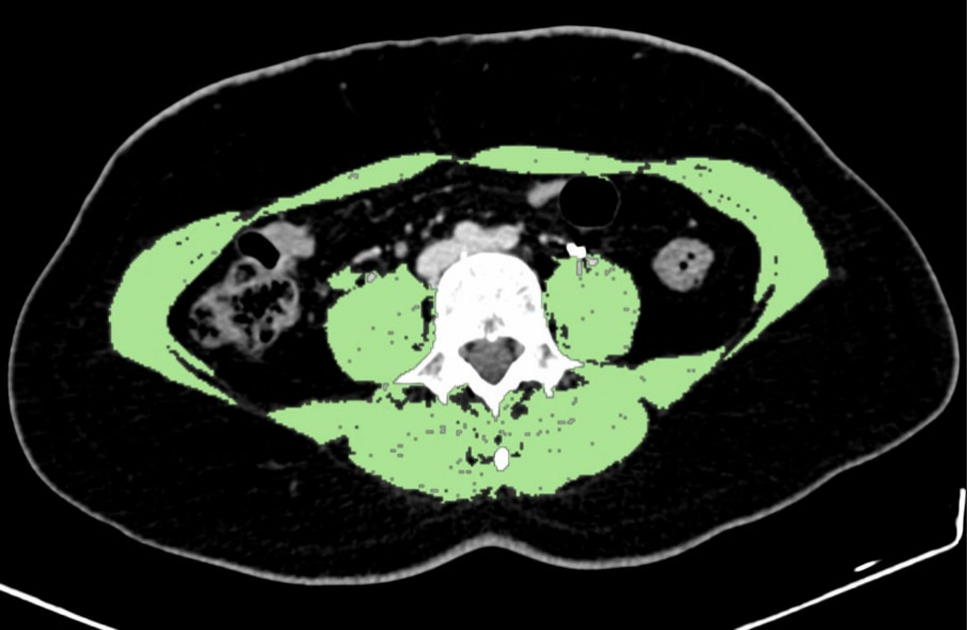
Axial CT image at the L3 vertebral level showing segmentation of abdominal skeletal muscles

The total abdominal muscle area was then divided by the square of the patient’s height to calculate the skeletal muscle mass index (SMI). Sarcopenia was defined using SMI cut-off values of ≤ 41 cm^2^/m^2^ for patients with a body mass index (BMI) of ≥ 25 and ≤ 43 cm^2^/m^2^ for those with a BMI of < 25 [16] (Fig. 2).

### Endovascular Treatment

Endovascular treatment was performed by two interventional radiologists with more than 10 years of experience. Following local anesthesia, the right common femoral vein was punctured under ultrasound (US) guidance. A 6-Fr introducer was placed, followed by the advancement of a 5-Fr Cobra catheter (Terumo, Europe) and a 5-Fr Simmons1 catheter (Cordis Johnson & Johnson, Europe NV, Roden, Netherlands) through a 0.035 hydrophilic wire (Glidewire, Terumo Europe) to selectively catheterize each renal vein. Venographs were obtained during the Valsalva maneuver.

The ovarian veins exhibiting signs of insufficiency were superselectively catheterized with a 2.7-Fr microcatheter (Renegade, Boston Scientific, USA) and embolized using metallic coils, vascular plugs, and 5% Aethoxysklerol in foam form. Following the procedure, patients were discharged on the same day with appropriate recommendations.

### Pain Assessment

The pain levels of the patients were assessed using the Visual Analog Scale (VAS). Pain scores were reassessed using the same questionnaire prior to treatment and at the third, sixth, and twelfth months post-procedure. Treatment success was defined as a reduction in pain levels of 50–100%, while treatment failure was defined as a reduction of less than 50% [17].

## Statistical Analysis

Patients who underwent endovascular treatment for PVI were divided into groups based on the presence of sarcopenia. The demographic data, radiological findings, and clinical outcomes of these groups were analyzed and compared. Descriptive statistics were presented as percentages and frequencies for categorical variables, and as means and standard deviations for continuous variables.

The chi-square test and Fisher’s exact chi-square test were used to compare qualitative data between groups. Quantitative data were tested for normal distribution and homogeneity prior to analysis. Variables with a normal distribution were compared using Student’s t-test, while non-normally distributed variables were analyzed using non-parametric tests, if applicable. The correlation between sarcopenia and treatment outcomes was evaluated using Pearson correlation.

A *p*-value < 0.05 was considered statistically significant. Statistical analysis was performed using TURCOSA (Turcosa Analitik Ltd. Co., Turkey).

## Results

In this study, a total of 62 patients with a mean age of 45 ± 17.9 years were evaluated. According to their skeletal muscle mass index (SMI), 22 patients (35.5%) were identified as sarcopenic. The demographic and clinical characteristics of the patients treated for pelvic venous insufficiency (PVI) are presented in Table 1.

**Table 1:** Demographic and clinical characteristics of patients treated for pelvic venous insufficiency

Variable	Mean	Range (LL-UL)
Age (years)	45.7 ± 17.9	27–65
Height (cm)	163.7 ± 6.3	150–178
BMI (kg/cm^2^)	29.13 ± 5.3	19.4–44.4
TAMA	120.8 ± 15.2	92.4–160.8
SMI	45.2 ± 6.7	32.5–71.4
Preprocedural VAS score	35.8 ± 16.5	6–82
Postprocedural third-month VAS score	13.41 ± 10.9	0–46
VAS score change (%)	− 62.95 ± 23.8	− 13.6 to − 100

SD: Standard deviation; UL: Upper limit; LL: Lower limit; BMI: Body mass index; VAS: Visual Analog Scale; TAMA: Total abdominal muscle area; SMI: Skeletal muscle index

When the patients were stratified into groups based on the presence of sarcopenia, there were no significant differences in the pre-procedural and post-procedural third-month Visual Analog Scale (VAS) scores between the sarcopenic and non-sarcopenic groups (36.2 ± 16.9 vs. 34.9 ± 16.1, *p* = 0.781, and 12.1 ± 10.8 vs. 16.1 ± 11.2, *p* = 0.196, respectively). However, the reduction in VAS scores post-treatment was significantly greater in non-sarcopenic patients (67.6 ± 22.4%) compared to sarcopenic patients (53.1 ± 24.4%, *p* = 0.025). Clinical success was achieved in 54 out of 62 patients (87.1%). In the non-sarcopenic group, all patients (42/42, 100%) experienced clinical success, whereas clinical success was achieved in 12 out of 20 patients (60%) in the sarcopenic group. This difference was statistically significant (*p* < 0.001).

There were no significant differences between the groups in terms of the procedural materials and techniques used (Table 2).

**Table 2:** Comparison of demographic and clinical findings between non-sarcopenic and sarcopenic groups in patients treated for pelvic venous insufficiency

Variable	Non-Sarcopenic group(n = 42)	Sarcopenic group (n = 20)	*p*-value
Age (years)	47.6 ± 21.1 (41.1–54.2)	41.8 ± 6.7 (41.1–54.2)	0.231
Height (cm)	162.1 ± 5.3 (160.4–163.7)	167.4 ± 6.7 (164–170.5)	0.002
Ovarian vein diameter (mm)	7.7 ± 1.8 (7.1–8.3)	8.2 ± 2.1 (7.2–9.2)	0.366
Duration of illness (years)	14.5 ± 8.7 (1–25)	15,7 ± 6.5 (5–30)	0.261
Parity Status Low multi-parite Grand multi-parite	38 4	18 2	0.638
Dyspareunia (n)	25	12	0.597
The treated ovarian vein (Bilateral/Unilateral)	26/16	10/10	0.419
Processing Material (Coil/Others)	30/12	17/3	0.346
BMI	30.8 ± 5.3 (29.1–32.4)	25.6 ± 5.3 (29.2–32.5)	< 0.001
TAMA	126.8 ± 13.3 (122.6–131.1)	108.2 ± 9.8 (103.6–112.8)	< 0.001
SMI	48.3 ± 5.7 (46.6–50.1)	38.6 ± 2.7 (37.4–39.9)	< 0.001
Preprocedural VAS score	36.2 ± 16.9 (30.9–41.5)	34.9 ± 16.1 (27.4–42.5)	0.781
Postprocedural third-month VAS score	12.1 ± 10.8 (8.8–15.5)	16.1 ± 11.2 (10.8–21.3)	0.196
VAS score change (%)	− 67.6 ± 22.4 (− 60.6 to 74.6)	− 53.1 ± 24.4 (− 41.7 to 64.6)	0.025
Clinical Success (%)	42 (100)	12 (60)	< 0.001

SD: Standard deviation; LL: Lower limit; UL: Upper limit; *P*: P value; BMI: Body mass index; VAS: Visual Analog Scale; TAMA: Total abdominal muscle area; SMI: Skeletal muscle index

When patients were categorized based on the reduction in VAS scores post-treatment, successful treatment was achieved in 42 patients. A moderate negative correlation was found between the presence of sarcopenia and successful treatment outcomes (*r* = − 0.365, *p* = 0.004).

## Discussion

The findings of this study highlight the potential impact of sarcopenia on the clinical outcomes of patients undergoing embolization for pelvic venous insufficiency (PVI). Although both sarcopenic and non-sarcopenic patients demonstrated improvement in pain relief post-treatment, the degree of improvement differed significantly between the groups. This suggests that baseline muscle health may play a role in modulating treatment outcomes. Recognizing these differences is crucial for tailoring therapeutic approaches and identifying patients who might benefit from adjunctive interventions to optimize recovery.

Existing literature suggests that CPP may be associated with body habitus. Silva et al. [18] reported no significant relationship between obesity and endometriosis, another cause of CPP. Similarly, in the present study, we found no evidence to suggest that obesity affects PVI outcomes. Some researchers have hypothesized that obesity might offer a protective effect against venous insufficiency due to the compressive force exerted by dense abdominal fat on venous structures, isolating them from external compressive forces [19]. Supporting this hypothesis, BMI was higher in the non-sarcopenic group in our study, and these patients showed greater improvement in VAS scores following treatment.

Numerous studies in the literature have explored the association between pelvic pain and musculoskeletal disorders in women [20–22]. A common finding in these studies is that musculoskeletal dysfunction is often both a primary cause and a secondary consequence of CPP. It has been reported that 75% of patients with CPP have musculoskeletal disorders, and 85% exhibit symptoms such as postural changes, myofascial syndrome, and pelvic muscle contraction problems, all of which indicate dysfunction in the musculoskeletal system [20–22].

As demonstrated by Gurian et al. [21], pelvic muscle weakness, chronic spasm, and shortening of muscle length are frequently observed in women with CPP. These factors contribute to a loss of muscle synchrony, impaired rectal drainage, constipation, and the chronicity of CPP [23]. Similarly, muscle dysfunction can lead to impaired micturition, urinary retention, urgency, infections, and ultimately CPP [23]. Dysfunction in the pelvic floor muscles has also been identified as one of the most important causes of dyspareunia [24, 25].

Sarcopenia alone can result in all of the aforementioned symptoms, which are also observed in PVI, due to muscle weakening and dysfunction, as well as pelvic floor disorders [26]. From this perspective, sarcopenia should be considered alongside PVI in patients presenting with typical symptoms, and treatment plans should incorporate this consideration. As observed in our study, if appropriate nutritional and physiotherapeutic interventions are not implemented in patients with coexisting PVI and sarcopenia, optimal treatment outcomes are unlikely to be achieved.

This study has several limitations. Firstly, its retrospective design and the absence of a control group limit the ability to establish causality. Additionally, the relatively small sample size may affect the generalizability of the findings. The study relied solely on VAS as the primary outcome measure, which, while standardized and widely accepted, may not fully capture the multidimensional aspects of PVI and its impact on patients’ quality of life. Future studies should incorporate additional clinical and quality-of-life metrics to provide a more comprehensive evaluation of treatment outcomes.

This study focused exclusively on ovarian veins as the primary site of venous insufficiency. Other pelvic venous plexuses, such as uterine or internal iliac veins, were not evaluated. Expanding the scope to include these plexuses in future research would enhance the understanding of PVI management and outcomes. Additionally, dual-energy X-ray absorptiometry (DEXA), which is considered the gold standard for assessing muscle mass, was not utilized. Instead, body CT was employed to diagnose sarcopenia, which, while sensitive, may introduce variability. Myosteatosis—a condition characterized by lipid infiltration into inter- and intramuscular compartments—was also not assessed, which might have influenced treatment outcomes.

Finally, different embolic agents were used during the procedures, including coils, sclerotherapy, and plugs. Although the variation in embolic techniques was not statistically significant, its potential impact on treatment outcomes cannot be completely excluded.

CPP and PVI are significant health concerns for women, but they can be effectively managed with endovascular treatment. Recent studies have further emphasized the efficacy and safety of endovascular interventions in various patient populations, including those with chronic pelvic pain and nulliparous women [27, 28]. However, the potential presence of sarcopenia, frequently encountered in PVI patients, warrants further investigation. The management of sarcopenia should be incorporated into the overall treatment plan for PVI. In this study, unlike the relationship between varicoceles and PVI, no association was found between obesity and PVI. Future research involving larger patient cohorts, control groups, and extended follow-up periods is essential to better elucidate the relationships among PVI, sarcopenia, and obesity.

## Data Availability

The patient data used in the study are available in our hospital's PACS and patient information system.
